# CCL18 from tumor-associated macrophages promotes angiogenesis in breast cancer

**DOI:** 10.18632/oncotarget.5325

**Published:** 2015-09-23

**Authors:** Ling Lin, Yong-Song Chen, Yan-Dan Yao, Jing-Qi Chen, Jia-Ning Chen, Song-Yin Huang, Yun-Jie Zeng, He-Rui Yao, Si-Hai Zeng, Yong-Shui Fu, Er-Wei Song

**Affiliations:** ^1^ Guangdong Provincial Key Laboratory of Malignant Tumor Epigenetics and Gene Regulation, Medical Research Center, Sun Yat-Sen Memorial Hospital, Sun Yat-Sen University, Guangzhou 510120, P. R. China; ^2^ Breast Tumor Center, Sun Yat-Sen Memorial Hospital, Sun Yat-Sen University, Guangzhou 510120, P. R. China; ^3^ Department of Internal Medicine, the First Affiliated Hospital, Shantou University Medical College, Shantou 515041, P. R. China; ^4^ Department of Laboratory, Sun Yat-Sen Memorial Hospital, Sun Yat-Sen University, Guangzhou 510120, P. R. China; ^5^ Department of Pathology, Sun Yat-Sen Memorial Hospital, Sun Yat-Sen University, Guangzhou 510120, P. R. China; ^6^ Department of Oncology, Sun Yat-Sen Memorial Hospital, Sun Yat-Sen University, Guangzhou 510120, P. R. China; ^7^ Guangzhou Blood Center, Guangzhou 510120, P. R. China

**Keywords:** breast cancer, tumor-associated macrophage, angiogenesis, CCL18, PITPNM3

## Abstract

The infiltration of tumor-associated macrophages (TAMs) is associated with extensive angiogenesis, which contributes to a poor prognosis in breast cancer. However, anti-angiogenic therapy with VEGF-specific monotherapy has been unsuccessful in treating breast cancer, and the molecular mechanisms associated with chemoresistance remain unclear. Here, we investigated whether CCL18, a chemokine produced by TAMs, can stimulate angiogenesis in breast cancer, as well as the underlying mechanisms. Double immunohistochemical staining for CCL18 and CD34/CD31/vWF was performed in 80 breast cancer samples to study the correlation between CCL18^+^ TAMs and microvascular density (MVD). Cocultures of TAMs with human umbilical vein endothelial cells (HUVECs) were used to model the inflammatory microenvironment, and CCL18-induced angiogenesis was evaluated both *in vitro* and *in vivo*. We demonstrated that CCL18^+^ TAM infiltration positively associated with MVD in breast cancer samples, which was correlated with tumor metastasis and poor prognosis. We confirmed, both *in vitro* and *in vivo*, that CCL18 and VEGF synergistically promoted endothelial cell migration and angiogenesis. Conversely, blocking CCL18 or VEGF with neutralizing antibodies synergistically inhibited the promigratory effects of TAMs. Silencing PITPNM3, a putative CCL18 receptor, on the surface of HUVECs abrogated CCL18-mediated promigration and the enhancement of HUVEC tube formation, independently of VEGFR signaling. Moreover, CCL18 exposure induced the endothelial-mesenchymal transformation and activated ERK and Akt/GSK-3β/Snail signaling in HUVECs, thereby contributing to its pro-angiogenic effects. In conclusion, our findings suggest that CCL18 released from TAMs promotes angiogenesis and tumor progression in breast cancer; thus, CCL18 may serve as a novel target for anti-angiogenic therapies.

## INTRODUCTION

Angiogenesis, the formation of new blood vessels, facilitates tumor growth, progression, and aggressiveness [1]. Numerous studies have reported a correlation between increased angiogenesis and poor prognosis in various cancers, such as breast, prostate, gastrointestinal, cervical, uterine, and lung cancer; thus, inhibiting angiogenesis is potentially a promising strategy for numerous cancer therapies [2, 3].

Various cytokines contribute to tumor angiogenesis, including vascular endothelial growth factor (VEGF), basic fibroblast growth factor, epidermal growth factor, interleukin (IL)-8, and tumor necrosis factor-α [4, 5]. Among them, VEGF is the primary cytokine that promotes angiogenesis in solid tumors by promoting endothelial cell proliferation, migration, and vascular permeability. Thus, anti-VEGF therapies are efficacious in treating several cancer types [6]. Indeed, the combination of anti-angiogenic therapy with conventional therapies, in particular radiation therapy and cytotoxic chemotherapy, has led to significant increases in overall survival in certain cancers such as colorectal carcinoma, metastatic renal cell carcinoma, non-squamous non-small cell lung cancer, and recurrent glioblastoma [4–6].

However, limitations exist with anti-angiogenic therapy. For example, bevacizumab, a humanized mouse monoclonal antibody to VEGF was unsuccessful in the treatment of breast cancer due to acquired resistance [7]. Recent data suggested that this dichotomy may stem from intrinsic differences in the stromal component of different cancers; however, the molecular mechanisms remain unclear [8].

The tumor microenvironment polarizes macrophages toward M2 or a mixed M1/M2 phenotype, which is characterized by elevated expression of potent pro-angiogenic factors [9–11]. Tumor-associated macrophage (TAM) infiltration is associated with vascular density and poor relapse-free and overall survival in various human malignancies, including breast cancer [2, 3, 12, 13]. The ability of TAMs to accelerate vessel growth is mediated by increased secretion of several pro-angiogenic factors. Therapeutic success in blocking these pro-tumor activities in preclinical models and early clinical trials suggests macrophages as effective targets for combination cancer therapy [14].

Results from our previous study revealed that CCL18, the most abundant and specific chemokine produced by TAMs in breast cancer stroma, promotes adherence to the extracellular matrix and enhances the invasiveness of breast cancer cells [15]. We hypothesized that CCL18 may also be involved in tumor angiogenesis and promote resistance to anti-VEGF monotherapy. Therefore, we evaluated the role of CCL18 released from TAMs in promoting angiogenesis in breast cancer tissues.

## RESULTS

### Correlation of CCL18 expression in breast TAMs with tumor angiogenesis

**Figure 1:**
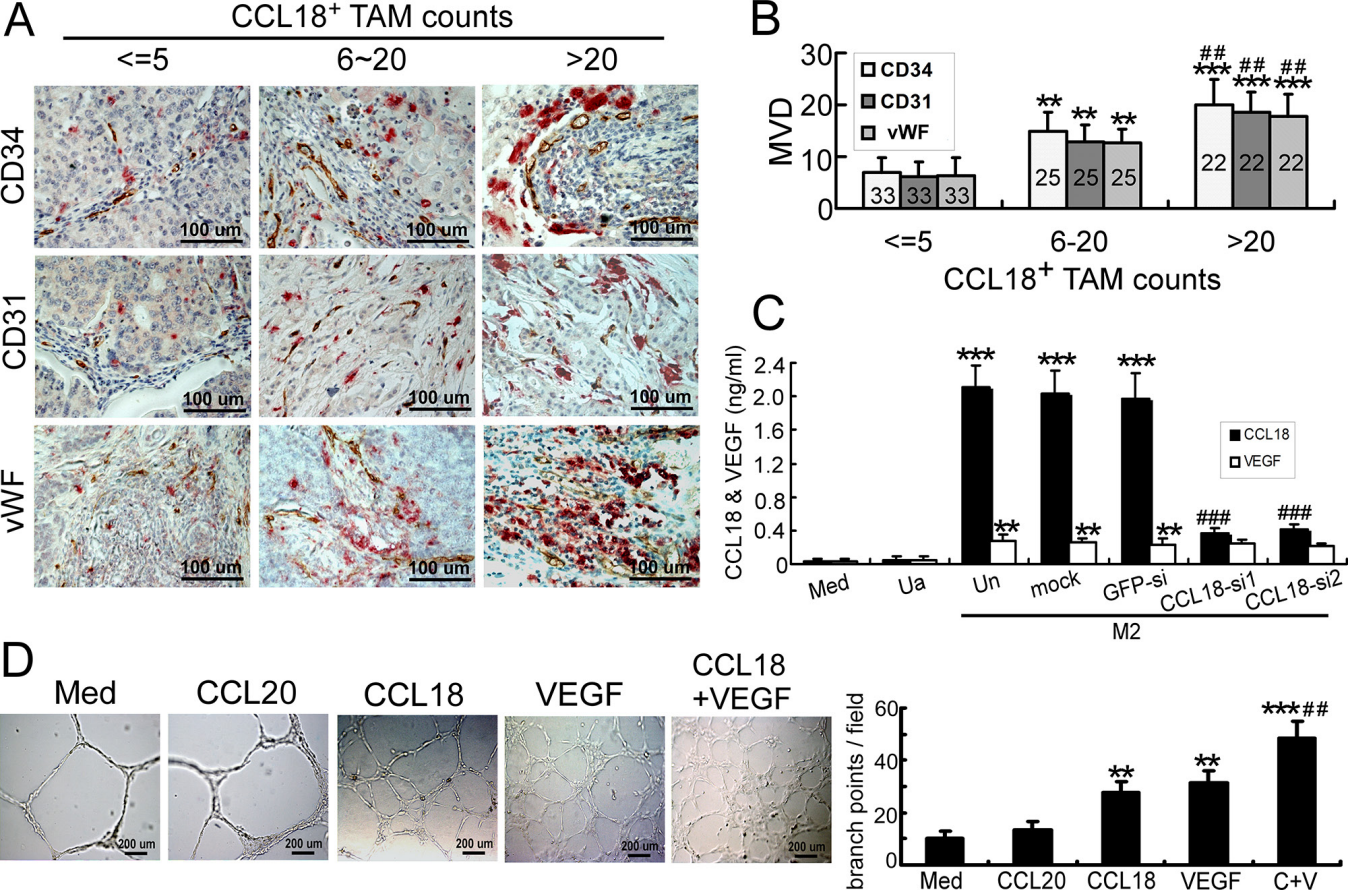
Correlation of CCL18 expression in breast TAMs with tumor MVD **A.** Representative double-IHC staining of TAMs for CCL18^+^ (red) and either CD34, CD31, or vWF (brown) in human breast invasive ductal carcinoma samples with low (≤ 5 per view of field; *n* = 33), medium (6–20 per view of field; *n* = 25), or high (> 20 per view of field; *n* = 22) CCL18^+^ TAM counts. Scale bar, 100 μm. **B.** MVD quantification as determined by CD34/CD31/vWF IHC staining in human breast invasive ductal carcinoma samples with low, medium, or high CCL18^+^ TAM counts. Bars correspond to means ± SEMs. The number of samples in each group is indicated. **p < 0.01 versus low CCL18^+^ cell counts; ***p < 0.001 versus low CCL18^+^ cell counts; ^##^*p* < 0.01 versus medium CCL18^+^ cell counts. **C.** IL-4-activated monocyte-derived macrophages (MDMs) were obtained by growing PBMCs in culture medium containing 45 ng/mL rIL-4 in 24-well culture plates for 7 days. Unactivated MDMs were prepared similarly, but grown in the absence of rIL-4. Afterward, IL-4-activated MDMs were transfected with GFP or CCL18 siRNAs. Expression levels of the CCL18 and VEGF cytokines were measured by ELISAs using supernatants from unactivated MDMs (Ua) or IL-4-activated MDMs (M2), which were untransfected (Un), mock-transfected, or transfected with either of 2 CCL18-siRNAs or a GFP-siRNA. Bars correspond to means ± SEMs from 5 independent experiments. **p < 0.01 and ***p < 0.001 versus medium; ^###^*p* < 0.001 versus untransfected M2 (Un). **D.** Representative images of Matrigel tube-formation assays in HUVECs treated with rCCL18 (20 ng/mL), rCCL20 (20 ng/mL), rVEGF (10 ng/mL), or a combination of rCCL18 (20 ng/mL) and rVEGF (10 ng/mL). Quantitative analysis of tube formation was performed by measuring the branch points of tubular structures formed. Scale bar, 200 μm. Bars correspond to means ± SEMs from 3 independent experiments. **p < 0.01 and ***p < 0.001 versus the medium group; ^##^*p* < 0.01 versus the rCCL18-treated group.

To investigate the correlation between CCL18 expression and tumor angiogenesis, we performed double immunohistochemical (IHC) staining for CCL18 expression using anti-CCL18 antibodies and microvascular density (MVD) using anti-CD34/CD31/von Willebrand Factor (vWF) antibodies in 80 primary invasive ductal carcinoma samples of the breast. MVD was measured as a marker to evaluate the occurrence of angiogenesis, since it was reported as an independent and highly significant prognostic factor for both node-negative and node-positive cancers [3, 16, 17]. Infiltration of high CCL18-expressing (CCL18^+^) TAMs correlated with high MVD (Figure 1A). Quantitatively, the MVD was proportional to the CCL18^+^ TAM count in breast cancer tissues (Table 1, Figure 1B). The association between CCL18^+^ TAM counts and the clinicopathological status of patients with breast cancer was then analyzed (Table 1). Consistent with our previous findings [15], the number of CCL18^+^ TAMs increased with higher tumor burden as defined by tumor size (*p* < 0.001) and staging (*p* = 0.014), as well as with aggressive tumor biology defined by advanced histological grading (*p* = 0.035), lymph node metastasis (*p* < 0.001), and distant metastasis (*p* = 0.018). Thus, CCL18 released by TAMs correlated with increased breast cancer angiogenesis, which may cause poor clinical outcomes in patients with breast cancer.

**Table 1:** Correlation of CCL18^+^ TAM counts with MVD and clinicopathological status in samples from 80 patients with breast invasive ductal carcinoma

CCL18^+^ TAM counts	≤ 5 (*n* = 33)	6–20 (*n* = 25)	> 20 (*n* = 22)	*p* value
**MVD**				
CD34	7.0	14.8	20.1	0.006
CD31	6.1	12.9	18.5	0.008
FVIII-ra	6.3	12.6	17.7	0.023
**Age (years)**				
≤ 45	16	9	8	0.545
> 45	17	16	14	
**Tumor Size (cm)**				
≤ 2	21	5	2	< 0.001
> 2	12	20	20	
**Stage**				
I	9	2	0	0.014
II	16	11	9	
III	8	12	13	
**Histological Grade**				
I	11	2	2	0.035
II	14	13	8	
III	8	10	12	
Distant metastasis*				
Positive	2	5	8	0.018
Negative	31	20	14	
**Lymph Node Metastasis**				
0	23	5	2	< 0.001
1–3	8	12	10	
≥ 4	2	8	10	
**ER status**				
Positive	23	19	17	0.784
Negative	10	6	5	
**Her2 status**				
Positive	3	5	8	0.047
Negative	30	20	14	

* Distant metastasis identified during postoperative follow-up consultation

Results from our previous study showed that among the cytokines released by breast TAMs, CCL18 is the most abundant [15]. VEGF is another pro-angiogenic cytokine released by TAMs in hypoxic avascular areas of breast cancers [4, 18]. Because breast TAMs are primarily M2 macrophages activated by Th2 cytokines, most notably IL-4 [19], we performed ELISAs to determine the cytokine levels of CCL18 and VEGF produced from IL-4-activated M2 macrophages. As shown in Figure 1C, although both CCL18 and VEGF were secreted, CCL18 levels were dramatically upregulated in IL-4-activated M2 cells. Furthermore, the specificity of CCL18 expression and production in IL-4-activated M2 cells was confirmed by transfecting the cells with small interfering RNAs (siRNAs) targeting CCL18 mRNA (Supplementary Figure S1; Figure 1C).

Because angiogenesis involves endothelial cells, we examined angiogenic responses in human umbilical vein endothelial cells (HUVECs). To investigate the effects of CCL18 on angiogenesis, we first performed Matrigel tube-formation assays, which are commonly used to assess the ability of endothelial cells to form 3-dimensional structures *in vitro*. The endothelial tubular structures formed by HUVECs treated with recombinant CCL18 (rCCL18) and rVEGF exhibited 2.7 and 3.1 times more branch points (both *p* < 0.01), respectively, than HUVECs treated with media alone (Figure 1D). Interestingly, the combined use of rCCL18 and rVEGF synergistically promoted the formation of tubular structures (*p* < 0.01 versus the CCL18 group; Figure 1D).

### Breast TAMs promoted HUVEC migration via CCL18

The stimulation of endothelial cell motility and proliferation is the initial event in the formation of new peritumoral blood vessels, which promotes tumor growth and survival [20]. Therefore, we tested whether CCL18 released by TAMs could induce migration in primary cultures of human endothelial cells and thus act as a cofactor in facilitating angiogenesis.

**Figure 2:**
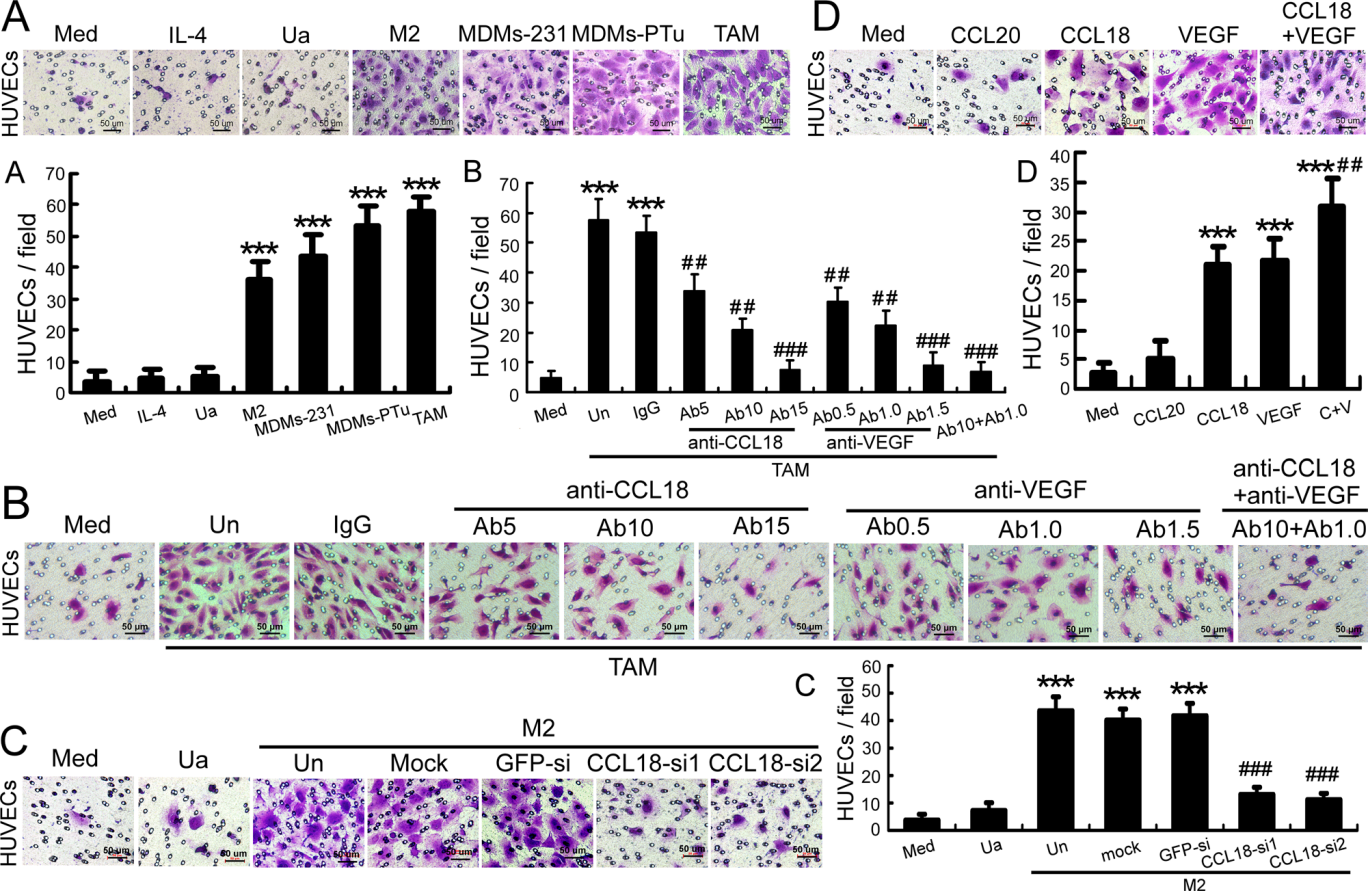
Breast TAMs and IL-4-activated MDMs promoted the migration of HUVECs via CCL18 **A.** Boyden chamber migration-assay results for HUVECs seeded into upper cell-culture inserts, with culture medium alone (Med), medium with IL-4 (IL-4), unactivated MDMs (Ua), IL-4-activated MDMs (M2), MDMs previously cocultured with MDA-MB-231 cells (MDMs-231) or primary breast cancer cells (MDMs-PTu), or primary breast TAMs (TAM) in the lower chambers. **B.** Boyden chamber migration-assay results for HUVECs seeded into upper cell culture inserts, with TAMs (TAM) plated in the lower chambers in the presence or absence of anti-CCL18 antibodies at 5 (Ab5), 10 (Ab10), or 15 μg/mL (Ab15); anti-VEGF antibodies at 0.5 (Ab0.5), 1.0 (Ab1.0), or 1.5 μg/mL (Ab1.5); combined anti-CCL18 (10 μg/mL) and anti-VEGF (1.0 μg/mL) antibodies (Ab10+Ab1.0); or an isotype-matched IgG control (IgG). **C.** Boyden chamber migration-assay results for HUVECs seeded into upper cell-culture inserts, with unactivated MDMs (Ua) or IL-4-activated MDMs (M2) plated in the lower chambers, which were untreated (Un), mock-transfected, or transfected with a GFP-siRNA or either of the 2 CCL18-siRNAs. **D.** Boyden chamber migration-assay results for HUVECs treated with rCCL18 (20 ng/mL), rCCL20 (20 ng/mL), rVEGF (10 ng/mL), or combined rCCL18 (20 ng/mL) and rVEGF (10 ng/mL) (C+V) added to culture medium in the lower chambers. (A–D) Scale bar, 50 μm. Bars correspond to means ± SEMs obtained from 3 independent experiments. ***p < 0.001 versus HUVECs treated with medium alone; ^##^*p* < 0.01 and ^###^*p* < 0.001 versus HUVECs cocultured with untreated TAMs (B), untreated MDMs (C), or HUVECs treated with rCCL18 (D).

A coculture system for HUVECs, breast cancer cells, and macrophages was employed to mimic the inflammatory tumor environment. Macrophages were freshly isolated from human breast cancer tissues (primary TAMs) [15] or derived from monocytes (monocyte-derived macrophages, MDMs) that were activated by IL-4 treatment, or coculture with MDA-MB-231 or primary breast cancer cells. HUVEC migration in the coculture system was examined in Boyden chambers. Compared with HUVECs in grown medium alone, HUVEC migration increased by nearly 17-fold (*p* < 0.001) following coculture with primary TAMs for 6 h (Figure 2A). Similarly, the number of migrated HUVECs increased by 10-fold (*p* < 0.001), 12-fold (*p* < 0.001), and 15-fold (*p* < 0.001), respectively, when cocultured with MDMs activated by IL-4, MDA-MB-231, or primary breast cancer cells (Figure 2A). A direct influence of IL-4 on HUVEC migration was ruled out by adding IL-4 alone to the lower chambers. Thus, the migration of HUVECs exposed to TAMs or activated MDMs was dramatically enhanced compared to that observed with HUVECs exposed to untreated MDMs or culture media alone, suggesting that mediators released by TAMs or activated MDMs promoted HUVEC migration. These results also indicated that IL-4-activated MDMs may resemble TAMs *in vitro*.

CCL18 is the most abundantly secreted cytokine in breast TAMs [15]; thus, we employed neutralizing anti-CCL18 antibodies and 2 CCL18 siRNAs to investigate the role of CCL18 in HUVEC migration. VEGF was used as a positive control since it is a prototypical pro-angiogenic mediator [21]. Interestingly, in the coculture system with breast TAMs and HUVECs, blocking the effect of CCL18 with neutralizing anti-CCL18 antibodies inhibited TAM-dependent HUVEC migration in a concentration-dependent manner (*p* < 0.001; Figure 2B). Furthermore, combined treatment with neutralizing anti-CCL18 antibodies (10 μg/mL) and anti-VEGF antibodies (1.0 μg/mL) synergistically abolished the promigratory effects of TAMs to baseline levels (*p* < 0.001; Figure 2B). In addition, silencing CCL18 expression in IL-4-activated MDMs with either of 2 CCL18 siRNAs also significantly inhibited the chemotactic migration of HUVECs (*p* < 0.001; Figure 2C; Supplementary Figure S1), indicating that CCL18, a cytokine secreted from activated MDMs, does indeed stimulate HUVECs migration.

Next, we treated HUVECs with rCCL18, rVEGF, or rCCL18 and rVEGF in combination. The results showed that both CCL18 and VEGF enhanced HUVEC migration (both *p* < 0.001), and the combined use of both cytokines synergistically promoted endothelial cell migration (*p* < 0.01 versus the CCL18 group; Figure 2D). In contrast, treatment with rCCL20 (20 ng/mL), a member of the same chemokine family as CCL18, did not promote HUVEC migration (*p* > 0.05; Figure 2D). Collectively, these data suggested that CCL18 and VEGF from TAMs synergistically promoted the migration of endothelial cells.

**Figure 3:**
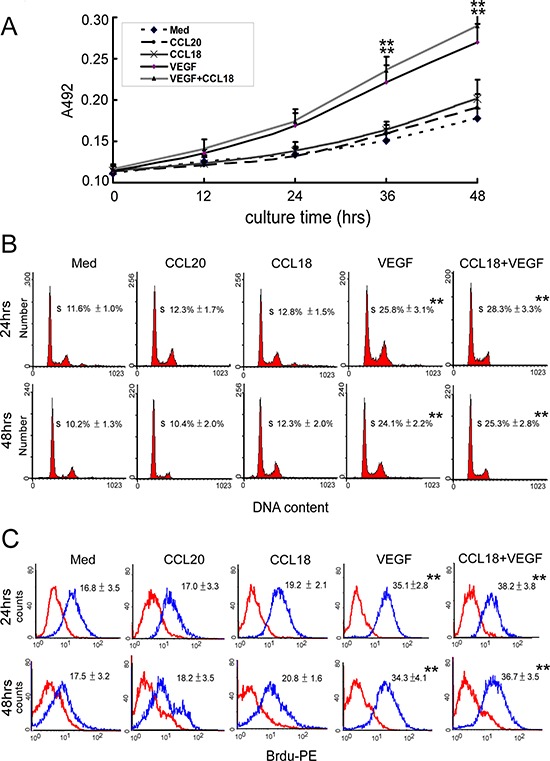
CCL18 promoted HUVEC angiogenesis without enhancing proliferation **A.** MTT assays for HUVECs cultured in media with or without rCCL18 (20 ng/mL), rCCL20 (20 ng/mL), rVEGF (10 ng/mL), or combined rCCL18 (20 ng/mL) and rVEGF (10 ng/mL) for 12, 24, 36, or 48 h. All values are means ± SEMs from 4 independent experiments. **p < 0.01 versus the media-only group. **B.** FACS analysis of cell cycle distribution in HUVECs cultured with or without rCCL18 (20 ng/mL), rCCL20 (20 ng/mL), rVEGF (10 ng/mL), or combined rCCL18 (20 ng/mL) and rVEGF (10 ng/mL) for 24 or 48 h. The histograms represent data from 4 independent experiments. All values are means ± SEMs from 4 independent experiments. **p < 0.01 versus the media-only group. **C.** BrdU-incorporation assays performed by flow cytometry in HUVECs treated as described in (B), using a PE-conjugated anti-BrdU antibody. The histograms represent 4 independent experiments. Means ± SEMs of fluorescence intensities for BrdU-treated HUVECs (blue) versus untreated cells (red) are indicated in each panel. **p < 0.01 versus the media-only group.

We further evaluated whether CCL18 may stimulate the proliferation of endothelial cells. Unlike VEGF, which stimulates endothelial cell proliferation during angiogenesis, incubating HUVECs with rCCL18 (20 ng/mL) for up to 48 h did not enhance cell proliferation compared with that for control cells, as determined in MTT assays (*p* > 0.05; Figure 3A), FACS analysis of cell cycle distribution (*p* > 0.05; Figure 3B), and bromodeoxyuridine (BrdU)-incorporation assays (*p* > 0.05; Figure 3C). Similarly, the combined use of rCCL18 and rVEGF did not synergistically promote HUVEC proliferation (all *p* > 0.05 versus the VEGF group; Figure 3A–3C).

### CCL18 enhanced the endothelial-mesenchymal transformation in HUVECs

**Figure 4:**
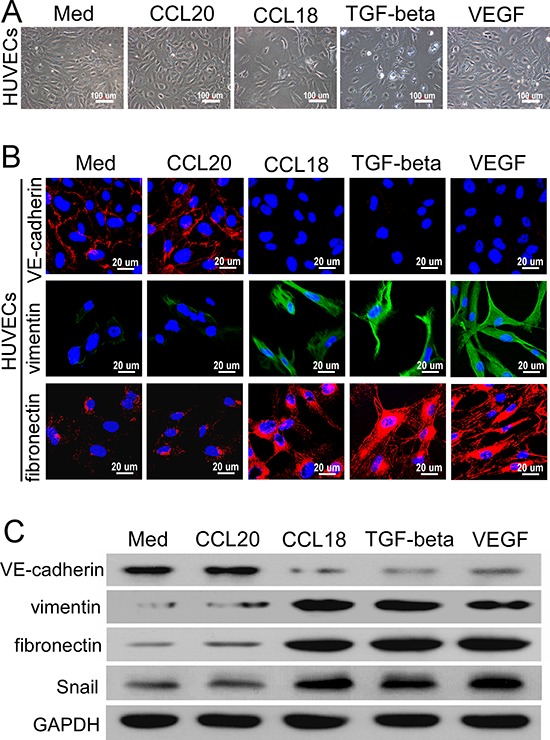
CCL18 enhanced the EndMT in HUVECs **A.** Phase-contrast microscopy images of HUVEC morphologies following treatment with rCCL18 (20 ng/mL), rCCL20 (20 ng/mL), TGF-β (10 ng/mL), or rVEGF (10 ng/mL) for 8 days. Scale bar, 100 μm. **B.** Confocal fluorescent microscopy images of VE-cadherin, vimentin, and fibronectin expression in HUVECs treated as described in (A). Cell nuclei were counterstained with DAPI. Scale bar, 20 μm. **C.** Representative western blots of VE-cadherin, vimentin, fibronectin, and Snail expression in HUVECs treated with rCCL18 (20 ng/mL), rCCL20 (20 ng/mL), TGF-β (10 ng/mL), or rVEGF (10 ng/mL). GAPDH was detected as a loading control.

Recent findings have demonstrated that endothelial cells at the angiogenic front may undergo endothelial-mesenchymal transformation (EndMT), which promotes angiogenic sprouting. EndMT is a specific form of the epithelial mesenchymal transition that can be induced by transforming growth factor (TGF)-β [22]. HUVECs were treated with rCCL18, rCCL20, VEGF, or TGF-β to investigate whether CCL18 induces EndMT in endothelial cells. As shown in Figure 4A, the morphology of HUVECs was altered dramatically from a typical cobblestone-like shape to an elongated, spindle-like shape after rCCL18 stimulation, resembling cells exposed to TGF-β. In addition, immunofluorescence staining demonstrated that vascular endothelial cadherin (VE-cadherin) expression was suppressed, while vimentin and fibronectin expression levels were dramatically increased upon treatment with rCCL18, VEGF, or TGF-β (Figure 4B); rCCL20 had no effect on the expression levels of these mesenchymal markers. In agreement with the immunofluorescence results, western blotting also demonstrated that treatment with rCCL18, VEGF, or TGF-β reduced the expression of VE-cadherin, but increased vimentin and fibronectin levels in HUVECs (Figure 4C). The expression of Snail, a transcriptional repressor associated with EndMT that is potentially induced by TGF-β [23, 24], was also enhanced following rCCL18 treatment (Figure 4C). Collectively, these data suggested that CCL18 induced EndMT in HUVECs, resulting in morphological and functional changes consistent with angiogenesis.

### Involvement of PITPNM3 in CCL18-stimulated HUVEC migration and tube formation

**Figure 5:**
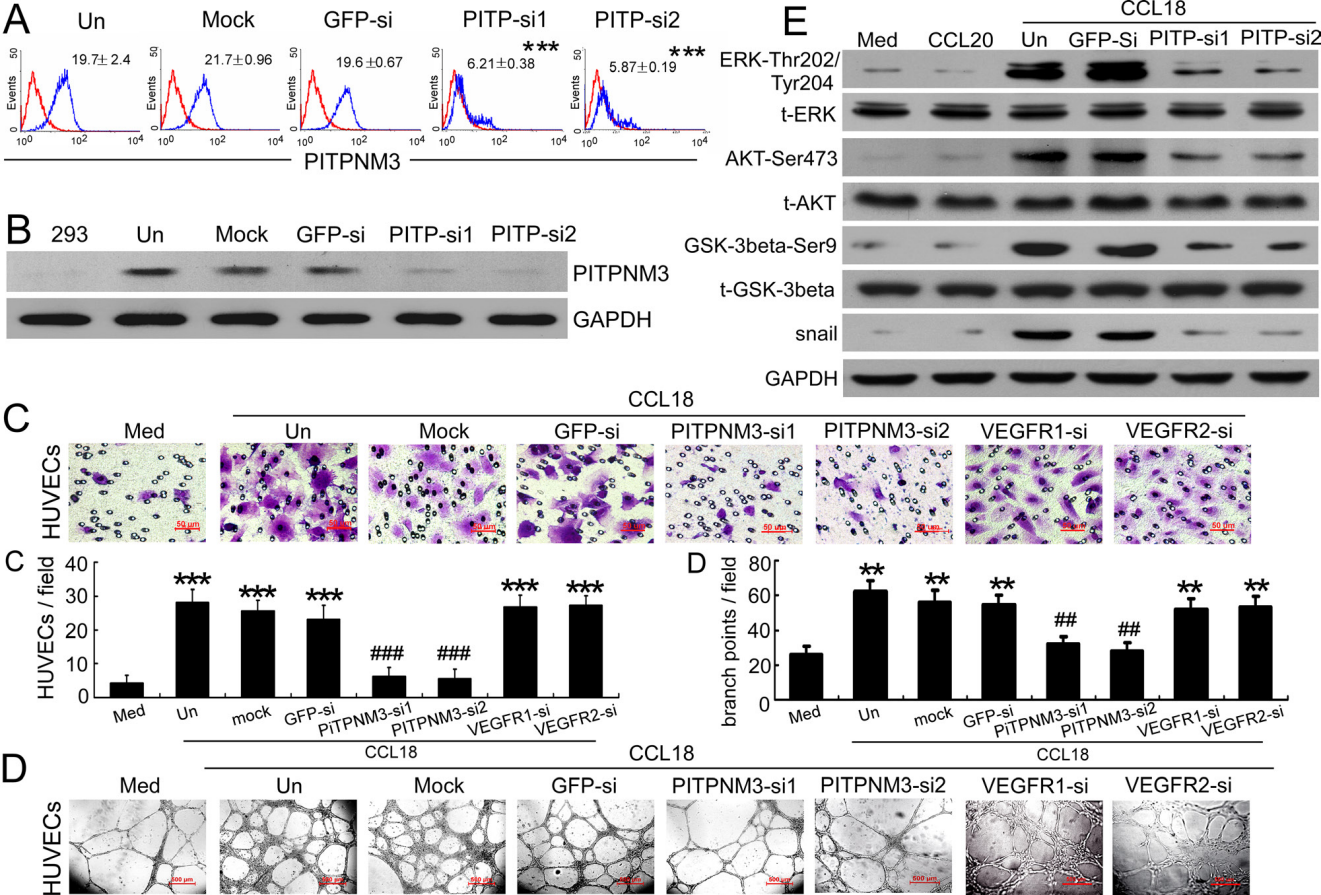
CCL18 enhanced HUVEC migration and tube formation via PITPNM3 **A.** Flow-cytometric analysis of PITPNM3 (PITP) expression (blue) relative to an IgG isotype control (red) in HUVECs that were untreated (Un), mock-transfected, or transfected with either of 2 PITPNM3 siRNAs or a GFP-siRNA. Mean fluorescence intensities ± SEMs for PITPNM3 immunostaining are indicated for 3 independent experiments. ***p < 0.001 versus untreated cells. **B.** Representative western blot results for PITPNM3 (PITP) expression in HEK293 cells and HUVECs transfected as described in (A). GAPDH was used as a loading control. **C–D.** Migration assays (C) and Matrigel tube-formation assays (D) in CCL18-treated HUVECs pretransfected with GFP, PITPNM3, VEGFR1, or VEGFR2 siRNAs. Scale bar, 50 μm. Bars correspond to means ± SEMs from 3 independent experiments. **p < 0.01 and ***p < 0.001 versus the media-only group; ^##^*p* < 0.01 and ^###^*p* < 0.001 versus the untransfected control (Un). **E.** Representative western blot results showing the phosphorylated and total levels of the ERK, AKT, GSK-3β, and Snail proteins in HUVECs treated with medium alone, rCCL20 (20 ng/mL), or rCCL18 (20 ng/mL), with or without transfection of either of 2 PITPNM3 (PITP) siRNAs or a GFP-siRNA. GAPDH was detected as a loading control.

Data from our previous studies showed that PITPNM3 is a putative receptor for CCL18 on the surface of breast cancer cells and that it mediates the effects of CCL18 by activating both the ERK and Akt/GSK-3β signaling pathways in cancer cells [15]. To elucidate the pro-angiogenic mechanism of CCL18, we evaluated whether CCL18 also promoted migration and tube formation in HUVECs via PITPNM3. Initially, the expression of PITPNM3 on HUVECs was tested by qRT-PCR, flow cytometry, and western blotting, and the specificity of PITPNM3 detection was confirmed using either of 2 PITPNM3 siRNAs (Supplementary Figure S2A; Figure 5A–5B).

To further investigate the pathophysiological relevance of the CCL18-PITPNM3 binding in HUVECs, we examined whether silencing of PITPNM3 influenced the biological effects of CCL18 on HUVECs. Our results show that transfection with either of 2 PITPNM3 siRNAs in HUVECs efficiently abrogated migration (*p* < 0.001; Figure 5C) and tube formation (*p* < 0.01; Figure 5D) induced by rCCL18. However, silencing VEGFR1 and VEGFR2 expression by transfecting HUVECs with VEGFR1-siRNA and VEGFR2-siRNA (Supplementary Figure S2B–S2C) did not suppress rCCL18-induced HUVEC migration or tube formation (both *p* > 0.05; Figure 5C–5D). These data indicated that CCL18 promoted HUVEC migration and tube formation via PITPNM3, independently of VEGFR signaling.

Several signal transduction pathways, including the PI3K/Akt/GSK-3β and MEK/ERK pathways, can mediate the promigratory functions of HUVECs [25]. Therefore, to explore the potential signaling pathways responsible for the angiogenic activity of CCL18 in HUVECs, western blot analysis was performed to determine the expression levels of phosphorylated ERK, Akt, and GSK-3β. Upon stimulation with rCCL18, ERK phosphorylation at Thr202/Tyr204, AKT phosphorylation at Ser473, and GSK-3β phosphorylation at Ser9 were markedly induced in HUVECs, whereas the total protein levels remained unchanged (Figure 5E). These results indicated a strong involvement of the PI3K/Akt/GSK-3β and MEK/ERK pathways in CCL18 receptor-mediated pro-migratory and pro-angiogenic activities in HUVECs. Furthermore, transfection with PITPNM3-siRNA abrogated the phosphorylation of ERK, Akt, and GSK-3β in HUVECs and downregulated Snail expression (Figure 5E), thus suggesting that CCL18 activated the ERK and Akt/GSK-3β/Snail signaling pathways via PITPNM3 in HUVECs, which may contribute to its pro-migratory effects and induction of EndMT.

### CCL18 promoted angiogenesis *in vivo*

**Figure 6:**
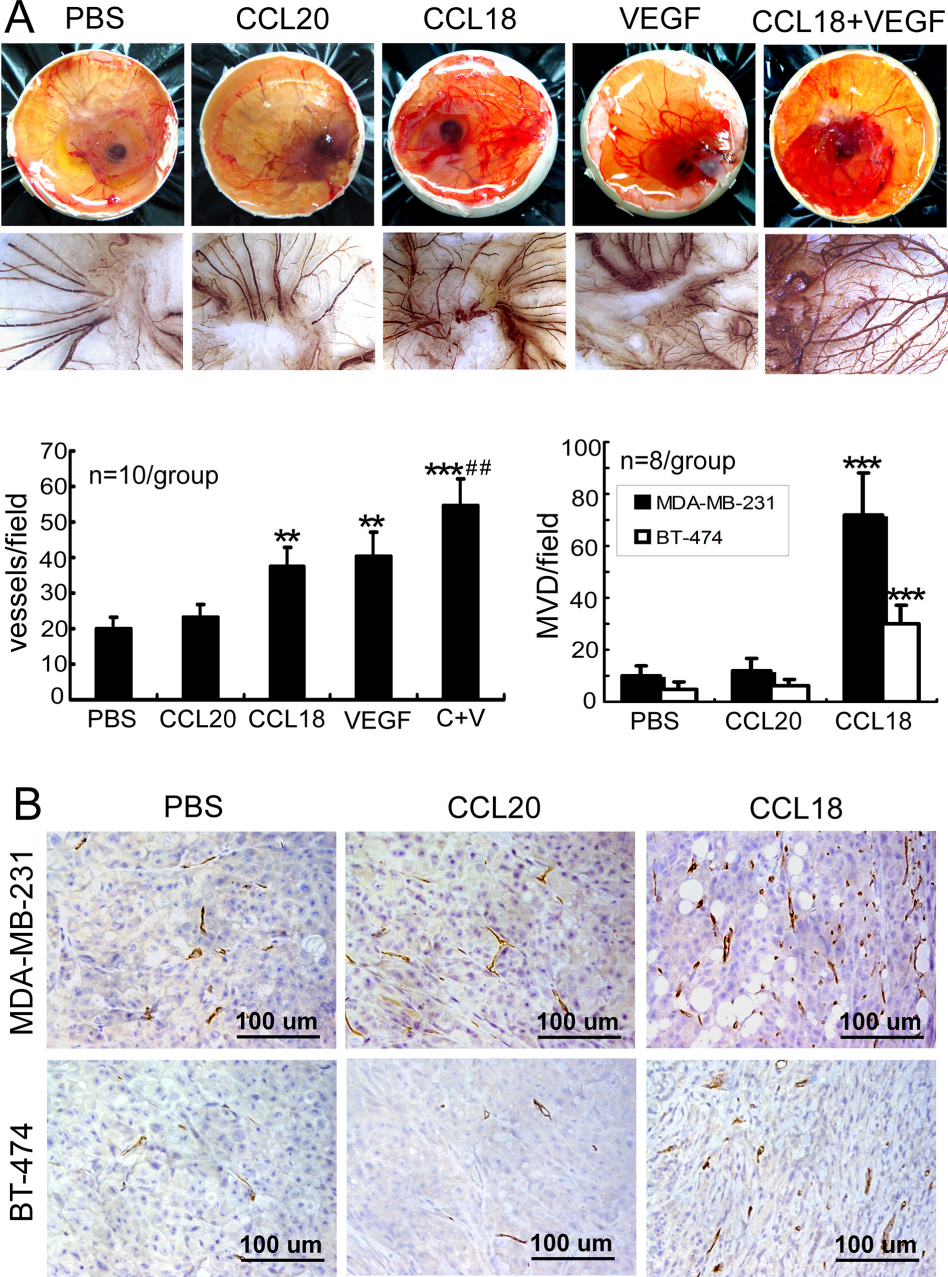
CCL18 promoted angiogenesis *in vivo* **A.** Stimulation of angiogenesis in CAMs treated with PBS, rCCL (20 ng/mL), rCCL18 (20 ng/mL), rVEGF (10 ng/mL), or a combination of rCCL18 (20 ng/mL) and rVEGF (10 ng/mL). Representative images of chicken embryos are shown (upper panels), and the small tortuous blood vessels were visible in CAMs. CAM tissues were then resected, and digital images were captured at 70× magnification (lower panels). Quantitative analysis of angiogenesis was performed by enumerating blood vessel formation in CAMs. Bars correspond to mean blood vessels per field ± SEMs. **p < 0.01 and ***p < 0.001 versus the PBS group; ^##^*p* < 0.01 versus the rCCL18 group. **B.** Determination of angiogenesis in breast cancer xenografts via MVD. Representative IHC staining of CD34 in mice bearing MDA-MB-231 or BT-474 xenografts following intratumoral injection with PBS, rCCL20, or rCCL18. Scale bar, 100 μm. Bars correspond to means ± SEMs. ***p < 0.001 versus the PBS group.

Finally, the angiogenic effects of CCL18 were confirmed *in vivo*. First, chicken chorioallantoic membrane (CAM) assays were performed to measure the formation of new blood vessels following treatment with rCCL18. Small tortuous blood vessels, a characteristic aspect of neovascularization, were visualized in the CAMs, and quantitative analysis revealed that treatment with rCCL18 or rVEGF, but not with rCCL20, significantly increased angiogenesis in CAMs compared to treatment with PBS (*p* < 0.01; Figure 6A). Similar to our *in vitro* findings, combined treatment with rCCL18 and rVEGF synergistically promoted angiogenesis in CAMs (*p* < 0.01 versus the rCCL18 group; Figure 6A).

To further examine whether CCL18 contributed to angiogenesis in breast cancer, we evaluated the angiogenic effects of CCL18, which does not have a mouse homolog [26], in tumor xenografts derived from MDA-MB-231 (high metastatic potential) or BT-474 (low metastatic potential) breast cancer cells in the mammary fat pads of NOD/SCID mice. Consistent with *in vitro* and CAMs findings, intratumoral injection with rCCL18, but not rCCL20, significantly increased the MVD counts by 7.2-fold (*p* < 0.001) in MDA-MB-231-derived xenografts and by 6.0-fold (*p* < 0.001) in BT-474-derived xenografts, as compared with control mice (Figure 6B). Taken together, our results suggested that CCL18 promotes angiogenesis in breast cancer.

## DISCUSSION

The tumor microenvironment is a complex ecology of cells that evolves with and provides support for tumor cells during the transition to malignancy. Clinical studies and experimental mouse models indicate that TAMs are generally tumorigenic. In primary tumors, macrophages can stimulate angiogenesis and enhance tumor cell invasion, motility, and intravasation. These protumoral macrophages not only increase the invasive capacity of tumor cells, but also increase the density of blood vessels, thereby increasing the number of circulating tumor cells and thus metastasis. Importantly, an anatomical structure consisting of macrophages, endothelial cells, and tumor cells, referred to as the tumor microenvironment for metastasis, is recognizable in histological sections and is predictive of metastasis with primary human breast cancers [27]. Therapeutic success in inhibiting these tumorigenic activities in preclinical models and in early clinical trials suggests that macrophages are attractive targets as part of combination therapy in cancer treatment [14].

Angiogenesis is also a complex physiological process involving multiple pathways that depend on homeostasis between stimulators and inhibitors. During the tightly controlled process of tumor angiogenesis, pro- and anti-angiogenic factors are produced by tumor and stromal cells in the tumor microenvironment [28]. TAMs constitute the most abundant immune cell population present in tumor microenvironments and can promote tumor progression and resistance to anticancer therapies (including anti-angiogenic therapies); thus, inflammation involving TAMs has been designated as the seventh hallmark of cancer [29–31]. A positive correlation between TAM infiltration and angiogenesis has been found in many human cancers, including breast cancer, melanoma, pulmonary adenocarcinoma, glioma, and gastric cancer [32–35]. In this study, we found that CCL18 immunostaining in TAMs was proportional to the MVD in breast cancer samples, which correlated with tumor metastasis and poor prognosis. Previously, we reported that CCL18 is a major cytokine released by TAMs and enhances breast cancer cell metastasis; moreover, CCL18 and CD68 colocalization in breast cancer tissues confirmed that CCL18-immunopositive cells represent a subset of macrophages [15].

Increasing evidence has demonstrated that resistance to VEGF-specific agents can be overcome by inhibiting a second pro-angiogenic pathway. These alternative angiogenic pathways are responsible for blood vessel growth and survival, even when the VEGF-pathway is blocked [36]. Most of these studies have also shown that simultaneous inhibition of VEGF and an alternative angiogenic factor improves therapeutic responses [37]. In the present study, we found that the pro-migratory and pro-angiogenic effects of CCL18 were independent of VEGFR signaling. Moreover, CCL18 and VEGF synergistically promoted the migration and angiogenesis of endothelial cells both *in vitro* and *in vivo*, and inhibition of CCL18 or VEGF with neutralizing antibodies synergistically blocked the pro-migratory effects of TAMs.

We further investigated possible molecular mechanisms that may be responsible for the pro-migratory effect of CCL18. In angiogenic endothelial cells, the Akt/GSK-3β signaling pathway promotes endothelial cell survival, migration, and capillary-like tube formation, which subsequently leads to angiogenesis [25]. Alternatively, ERK can stimulate tumor angiogenesis, and inhibition of ERK1/2 signaling in the tumor vasculature results in vessel reduction, defective vascular morphogenesis, and reduced tumor growth [38]. Chemokines exert their effects by binding to specific transmembrane G protein-coupled receptors. Our previous data revealed that CCL18 promotes extracellular matrix adherence and breast cancer cell invasion by binding to its potential cognate surface receptor PITPNM3, whose expression is essential for CCL18-induced calcium influx and chemotaxis [15]. In this study, the expression of PITPNM3 in HUVECs was verified by flow cytometry and western blot. Silencing PITPNM3 on the HUVEC cell surface abrogated CCL18-mediated migration enhancement and tube formation of HUVECs. Furthermore, the proangiogenic and promigratory effects of CCL18 were independent of VEGFR signaling. These data indicated that CCL18 promoted the HUVEC migration and tube formation via PITPNM3, independently of VEGFR signaling. These findings suggest a new intracellular pathway of CCL18-induced angiogenesis, implying that strategies which simultaneously inhibit CCL18 and VEGF signaling may improve the effects of anti-angiogenic therapies.

EndMT was first described in embryonic development during the formation of the heart valves. Recently, accumulating evidence has highlighted the critical role of EndMT during tumor angiogenesis [23, 24, 39]. For example, EndMT underlies angiogenic sprouting by enabling tip cells to promote emergence of the vascular plexus and migration into adjacent tissues [21, 22, 40]. Moreover, vascular support cells, such as pericytes and/or smooth muscle cells, may arise from the endothelia through EndMT [41]. Our data demonstrated that CCL18 induced EndMT in endothelial cells to produce a different differentiated phenotype, which may lead to loss of cell-cell junctions, as well as enhanced invasiveness and migratory capacities. We also found that Snail expression was upregulated by CCL18 via activation of the ERK and Akt/GSK-3β signaling pathways. Snail facilitates downregulation of VE-cadherin expression, which is essential for cell-cell binding and thus mediates EndMT [24, 42]. The induction of EndMT by CCL18 in endothelial cells, as observed in our study, should be considered an important preliminary observation meriting further investigation. In addition, the limited or non-existent effect of secreted CCL18 in stimulating HUVEC proliferation in this study agreed with observations from Ploenes et al. [43] and may have been due to the initiation of differentiating processes.

We further performed *in vivo* studies with CAMs, as well as breast cancer xenografts in NOD/SCID mice to assess the potential effects of CCL18 on angiogenesis. The results suggested that CCL18 promoted angiogenesis both *in vitro* and *in vivo*. In conclusion, our data describe a novel function of the versatile TAM-derived cytokine CCL18 in angiogenesis. Thus, CCL18 and/or its potential receptor PITPNM3 may serve as therapeutic targets for inhibiting angiogenesis in breast cancer, particularly for patients with resistance to anti-VEGF monotherapy.

To our knowledge, this is the first report implicating CCL18 in the promotion of tumor angiogenesis. Our findings suggest that inhibiting multiple pro-angiogenic pathways may sensitize tumors to anticancer therapies and help overcome resistance to anti-angiogenic therapies. Nevertheless, other angiogenic and anti-angiogenic peptides produced by TAMs and breast cancer cells may also contribute to tumor angiogenesis via different signaling pathways, thus, further investigations are needed to define the optimal targets and improve therapeutic responses to anti-angiogenic strategies.

## MATERIALS AND METHODS

### Ethic statement

All samples were collected with informed consent from patients, and all related procedures of investigation have been conducted in accordance with the ethical standards and according to the Declaration of Helsinki and according to national and international guidelines and have been approved by the review board of Sun Yat-Sen Memorial Hospital at Sun Yat-Sen University.

The protocols of all animal experiments were approved by the Animal Care and Use Committee of Sun Yat-Sen University and conformed to the legal mandates and national guidelines for the care and maintenance of laboratory animals. Surgery was performed under sodium pentobarbital anesthesia, and all efforts were made to minimize suffering.

### Patients and specimens

Formalin-fixed, paraffin-embedded tissue samples of primary invasive ductal carcinomas of the breast were obtained from 80 women (median age: 49.6 years, range: 29–76) at the Sun Yat-Sen Memorial Hospital, Sun Yat-Sen University, from January 2005 to October 2009. Pathological diagnoses were verified by 2 different pathologists.

### Double IHC staining

Double IHC staining of CCL18 and either CD34, CD31, or vWF was performed using the Doublestain System (Dako, Denmark) according to the manufacturer's protocols, using a rabbit polyclonal anti-CCL18 antibody (1:250 dilution; Sigma, USA), a mouse monoclonal anti-CD34 antibody (1:100 dilution; Abcam, USA), a mouse monoclonal anti-CD31 antibody (1:100 dilution; Dako), and a mouse monoclonal anti-vWF antibody (1:100 dilution; Abcam). Isotype-matched IgG was used as a negative control. The number of CCL18-positive cells per field of view was counted for at least 20 fields of view per section, at 40 × magnification. Small blood vessels were visualized by CD34/CD31/vWF staining of the endothelial cells. To determine the MVD, the highest neovascularization areas (hot spots) were identified by scanning whole tumor sections at low power, followed by counting individual microvessels at 400 × magnification. The highest single field value within each hot spot was recorded. Endothelial cells or endothelial cell clusters positive for CD34, CD31, or vWF that were separate from the adjacent clusters were considered as single countable microvessels [16].

### Cell culture and treatment

HUVECs were isolated from collagenase-digested umbilical veins and cultured in Human Endothelial Cell SFM (Gibco, USA) supplemented with 10% fetal calf serum (Gibco), endothelial cell growth supplement (60 μg/mL; BD, USA), and heparin (50 μg/mL; Sigma, USA) in an incubator with 5% CO_2_ at 37°C. Cells were used for the experiments between passages 3 and 6. MDA-MB-231 and BT-474 breast cancer cells and HEK293 embryonic kidney cells were obtained from the American Type Culture Collection (USA) and grown according to standard protocols. Peripheral blood mononuclear cells (PBMCs) from healthy donors were isolated by density-gradient centrifugation using Ficoll-Hypaque (Pharmacia, Sweden) [15]. For *in vitro* activation, PBMCs at a density of 1 × 10^6^ cells/mL were treated for 7 days with 45 ng/mL rIL-4 (R&D Systems, USA). Primary human TAMs and primary breast cancer cells were isolated from fresh breast cancer samples, as previously described [15, 26].

### Coculture experiments

Briefly, PBMCs were plated in the lower wells of 24-well Boyden chambers (Corning, USA) and cocultured for 7 days with MDA-MB-231 cells or primary breast cancer cells, which had been seeded on inserts having filter membranes with a 0.4-μm pore size. The pore size was sufficiently large to allow the passage of cytokines, but not direct cellular communication between the upper and lower chambers. Next, the upper chambers were removed and replaced with inserts having a pore size of 8 μm, containing plated HUVECs growing in Human Endothelial Cell SFM.

### ELISAs

CCL18 and VEGF levels in the supernatants of cultured macrophages were measured by performing ELISAs using a commercially available CytoSet kit (R&D, USA), as described by the manufacturer.

### Cell migration assay

We examined HUVEC migration using 24-well Boyden chambers (Corning, USA) containing 8-μM inserts coated with fibronectin (Roche, Germany), as described (15). HUVECs (1 × 10^5^ cells/well) were pretreated with mitomycin (10 μg/ml) for 2 h to block proliferation, after which they were plated in inserts in the upper chambers and cultured for 6 h in a humidified incubator at 37°C with 5% CO_2_. After 6 h, non-migrated cells at the top of the transwell filter were removed using a cotton swab. The invaded cells that migrated through the filter inserts were fixed in 4% paraformaldehyde and stained with 0.005% crystal violet (Sigma). The rates of migration were quantified as the number of cells per field of view using a phase-contrast microscope.

### siRNA preparation and transfection

HUVECs were plated at 5 × 10^5^ cells/ml in serum-free medium and transfected with siRNA duplexes using NeoFX Transfection Agent (Ambion, USA) according to the manufacturer's recommended protocol. The oligonucleotide sequences of the siRNAs used are as follows:

CCL18 siRNA-1, sense (5′-ACAAGUUGGUAC CAACAAATT-3′) and antisense (5′-UUUGUUGGUAC CAACUUGUGC-3′);

CCL18 siRNA-2, sense (5′-CCAGCAUUCUCA CUGUGAATT-3′) and antisense (5′-UUCACAGUGAGA AUGCUGGTT-3′);

PITPNM3 siRNA-1, sense (5′-GGGAGAAGUG GCUUCGUAATT-3′) and antisense (5′-UUACGAAGC CACUUCUCCCGG-3′);

PITPNM3 siRNA-2, sense (5′-CGCGCAUGAUC CUGCGCAATT-3′) and antisense (5′-UUGCGCAG GAUCAUGCGCGAG-3′);

VEGFR1 siRNA, sense (5′-CUGAGUUUAAA AGGCACCCdTdT-3′) and antisense (5′-GGGUGCCUU UUAAACUCAGdTdT-3′);

VEGFR2 siRNA, sense (5′-GGAAAUCUCUUG CAAGCUAUU-3′) and antisense (5′-UAGCUUGCAAG AGAUUUCCUU-3′); and

GFP siRNA, sense (5′-GGCUACGUCCAGGAGC GCACC-3′) and antisense (5′-UGCGCUCCUGGAC GUAGCCUU-3′).

### MTT assays

Cell proliferation was assessed in MTT assays. Briefly, cells were seeded in 96-well culture plates (2 × 10^3^ cells/well) and incubated in normal growth medium for 24 h. Subsequently, the cells were grown for an additional 12, 24, 36, or 48 h with or without tested condition media, i.e., rCCL18 (20 ng/mL), rCCL20 (20 ng/mL), rVEGF (10 ng/mL), or combined rCCL18 (20 ng/mL) and rVEGF (10 ng/mL). Next, 10 μl MTT solution (5 mg/ml; Sigma) was added to each well, and the plates were incubated for 4 h at room temperature. The growth medium was next replaced with 100 μl of DMSO per well, and the absorbance at 492 nm was measured. The results were evaluated by comparing absorbance values of the experimental wells with those of the control wells.

### Cell-cycle analysis

HUVECs were harvested by trypsinization, washed with ice-cold PBS, and frozen in ice-cold 70% ethanol for 4 h. Afterwards, the cells were resuspended with PBS and analyzed using a FACScan analyzer (Becton Dickenson, USA).

### BrdU incorporation assay

The rate of DNA synthesis was assessed in BrdU-incorporation assays to evaluate HUVEC proliferation in the indicated test media. BrdU (10 μM; Upstate, USA) was added to media for 24 or 48-h incubation periods. BrdU incorporation in the HUVECs was evaluated by immunostaining using a phycoerythrin (PE)-conjugated anti-BrdU antibody (Upstate) and analyzed by flow cytometry using a FACScalibur instrument with CellQuest Software (Becton Dickinson).

### Immunofluorescence staining

For immunofluorescence staining, the cells were incubated with primary antibodies against VE-cadherin (Cell Signaling Technology [CST], USA), vimentin (CST) or fibronectin (Abcam), followed by incubation with Alexa Fluor 488- or 555-conjugated secondary antibodies (Invitrogen, USA). For confocal microscopy, the cells grown on coverslips were counterstained with DAPI and were imaged using a confocal laser-scanning microscope (Carl Zeiss, Germany) with a core data acquisition system (Applied Precision).

### Western blot analysis

Protein extracts were resolved by 8%–15% SDS-PAGE, transferred to polyvinylidene fluoride membranes, and probed with antibodies against human VE-cadherin (CST), vimentin (CST), fibronectin (Abcam), Snail (CST), PITPNM3 (Santa Cruz, USA), and GAPDH (Santa Cruz), as well as against ERK, AKT, GSK-3β, and the corresponding phosphorylated proteins (CST). Peroxidase-conjugated anti-mouse or anti-rabbit IgGs (CST) were used as secondary antibodies, and the antigen-antibody reactions were visualized by enhanced chemiluminescence (ECL) assay (Thermo, USA).

### Cell surface flow cytometry

To evaluate the cell surface expression of PITPNM3 in HUVECs, the cells were detached at 4°C using PBS-EDTA, resuspended in calcium and magnesium-free PBS, and incubated at 4°C with an anti-PITPNM3 primary antibody (Santa Cruz) for 1 h. After washing with PBS, the cells were incubated with a secondary anti-rabbit, fluorescein-conjugated IgG antibody (Jackson ImmunoResearch, USA) at 4°C for 45 min. The cells were then resuspended in PBS and analyzed using a FACSCalibur instrument with CellQuest Software (Becton Dickinson).

### *In vitro* angiogenesis assay by tube formation

*In vitro* Matrigel tube-formation assays [44] were performed to analyze the pro-angiogenic effect of CCL18 on HUVECs. Briefly, Matrigel (BD, USA) was thawed on ice, plated into 96-well culture plates, and allowed to polymerize at 37°C for 60 min. HUVECs suspensions (1 × 10^4^ cells resuspended in 150 μl) were seeded on the polymerized Matrigel-coated surfaces. After 12 h of incubation at 37°C with tested condition media, the HUVECs aligned to form the cords that ultimately became the pattern for new capillary structures. The branch points of the tube structures were measured at 100 × magnification.

### *In vivo* angiogenesis assays using chicken CAMs

Fertilized chicken eggs were incubated at 37.8°C and a relative humidity of 80% in incubators rotating at intervals of 30 min. On incubation day 4, square windows were cut into the air chamber sides of the shells to expose the CAMs. Sterile paper filters (5-mm diameter) were placed on the surface of the CAMs in areas with minimal small blood vessels, and tested cytokines were added to these restricted areas. The windows in the shells were sealed with adhesive tape, and the eggs were further incubated for 48 h [45]. Representative CAMs from each group were photographed under a dissecting microscope, and the total number of vessels that had sprouted from the primary vessels of the CAMs was determined.

### Tumor xenografts

Female NOD/SCID mice (3 to 4 weeks old) were bred and maintained under defined conditions at the Animal Experiment Center of Sun Yat-Sen University. MDA-MB-231 and BT-474 breast cancer cells (2 × 10^6^) were injected into the mammary fat pads of mice. When the xenografts were palpable (~0.5 cm in diameter), intratumoral injection of PBS, rCCL20, or rCCL18 (0.1 μg/kg) was performed biweekly for 4 consecutive weeks. Tumor growth was evaluated by monitoring the tumor volume (TV = length × width^2^ × 0.5) every 3 days for 45 days. Then the animals were sacrificed and tumor xenografts of the mice were harvested for further evaluation. Cryosections (4 μm) of the tumor xenografts were stained with IHC for histological assessment.

### Statistical analysis

All statistical analyses were performed using SPSS version 13.0 for Windows (SPSS, USA). Mann–Whitney *U* tests or Kruskal–Wallis tests were used to compare categorical and continuous variables, and chi-squared tests or Fisher's exact tests were applied to compare 2 sets of categorical variables. All *in vitro* experiments were performed independently at least 3 experiments, with triplicate measurements for each sample. Differences with *p* values of less than 0.05 were considered statistically significant.

## SUPPLEMENTARY EXPERIMENTAL PROCEDURES, FIGURES


