# Supplementary material for: CCL18 from tumor-associated macrophages promotes angiogenesis in breast cancer
Source: Oncotarget. 2015 Sep 23;6(33):34758–73. doi: 10.18632/oncotarget.5325 (PMC4741488; doi:10.18632/oncotarget.5325)
Supplement: Supplementary file 1 [file oncotarget-06-34758-s001.pdf]

# SUPPLEMENTARY EXPERIMENTAL PROCEDURES

## qRT-PCR

Quantitative real-time reverse transcription PCR (qRT-PCR) was performed with a LightCycler 480 instrument (Roche Diagnostics, Switzerland), using the SYBR Premix Ex Taq TM (TaKaRa, Japan) according to the manufacturer's instruction. All reactions were done in a 25-µl reaction volume in triplicate. Primers for CCL18, PITPNM3, VEGFR1, VEGFR2, and GAPDH were obtained from Invitrogen. Following an initial denaturation step at 95°C for 30 s, 40 cycles of PCR amplification were performed at 95°C for 5 s and 60°C for 20 s. Standard curves were generated and the relative amount of target mRNA was normalized to that of GAPDH. The specificity was verified by melting curve analysis and agarose gel electrophoresis. The primer sequences were as follows:

| Gene | Forward primer | Reverse primer |
|---|---|---|
| CCL18 | 5′-CTCTGCTGCCTCGTCTATACCT-3′ | 5′-CTTGGTTAGGAGGATGACACCT-3′ |
| PITPNM3 | 5′-GAGAAGTGGCTTCGTAAGCGGA-3′ | 5′-TCTCTCCAGTCAGAGCCACCAT-3′ |
| VEGFR1 | 5′-GGTATCCCTCAACGTACA-3′ | 5′-CCACAGTCCCAACTTTATT-3′ |
| VEGFR2 | 5′-CCGTCAAGGGAAAGACTACG-3′ | 5′-AGATGCTCCAAGGTCAGGAA-3′ |
| GAPDH | 5′-ATCACCATCTTCCAGGAGCGA-3′ | 5′-CCTTCTCCATGGTGGTGAAGAC-3′ |

## SUPPLEMENTARY FIGURES

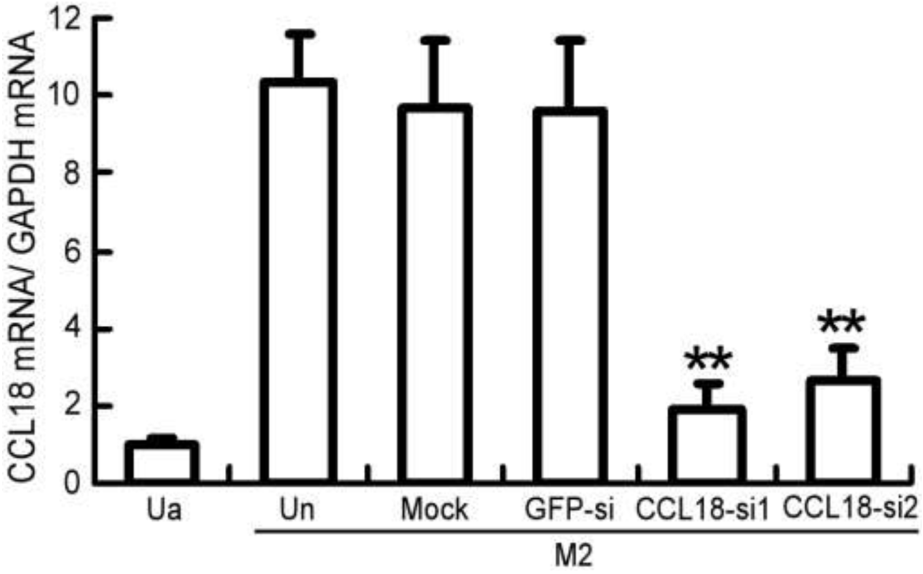


**Supplementary Figure S1: CCL18 mRNA expression in IL-4-activated monocyte-derived macrophages (MDMs).** qRT-PCR for CCL18 mRNA expression relative to GADPH mRNA in IL-4-activated MDMs (M2), which were untransfected (Un), mock-transfected (Mock), or transfected with GFP-siRNA or either of the 2 CCL18-siRNAs. Unactivated MDMs (Ua) were used as negative controls. Data were normalized to the group of unactivated MDMs (Ua). Bars correspond to means ± SEMs from 5 independent experiments. **$p < 0.001$ versus untransfected M2 (Un).

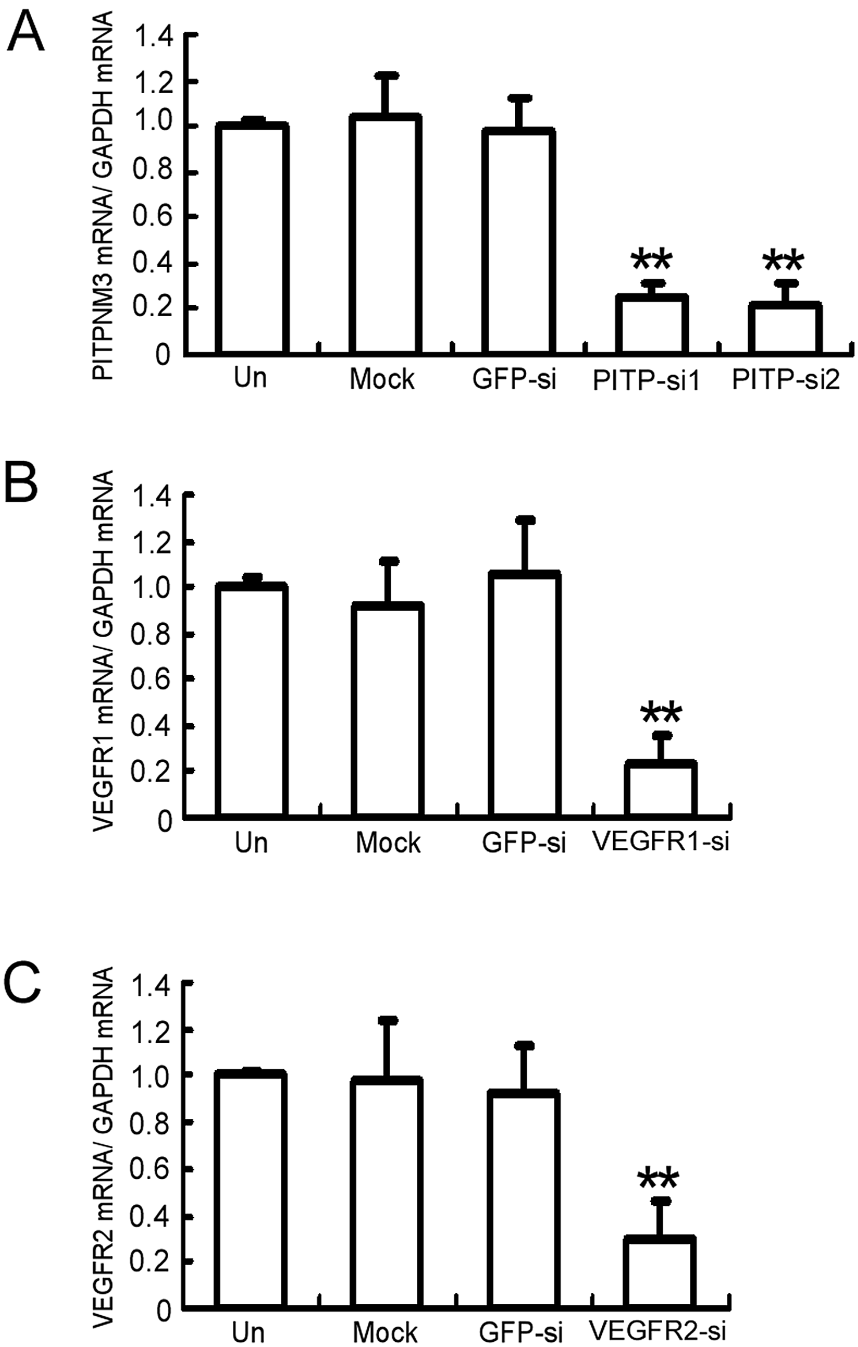


**Supplementary Figure S2: mRNA expression of PITPNM3, VEGFR1, or VEGFR2 in human umbilical vein endothelial cells (HUVECs). A.** qRT-PCR for PITPNM3 mRNA expression relative to GADPH mRNA in HUVECs, which were untransfected (Un), mock-transfected (Mock), or transfected with GFP-siRNA or either of the 2 PITPNM3(PITP)-siRNAs. Data were normalized to the group of untransfected HUVECs (Un). Bars correspond to means ± SEMs from 5 independent experiments. ** $p < 0.01$ versus untransfected HUVECs (Un). **B.** qRT-PCR for VEGFR1 mRNA expression relative to GADPH mRNA in HUVECs, which were untransfected (Un), mock-transfected (Mock), or transfected with VEGFR1-siRNA or GFP-siRNA. Data were normalized to the group of untransfected HUVECs (Un). Bars correspond to means ± SEMs from 5 independent experiments. ** $p < 0.01$ versus untransfected HUVECs (Un). **C.** qRT-PCR for VEGFR2 mRNA expression relative to GADPH mRNA in HUVECs, which were untransfected (Un), mock-transfected (Mock), or transfected with VEGFR2-siRNA or GFP-siRNA. Data were normalized to the group of untransfected HUVECs (Un). Bars correspond to means ± SEMs from 5 independent experiments. ** $p < 0.01$ versus untransfected HUVECs (Un).
